# Supplementary material for: Anti-SARS-CoV-2 Activity of Rhamnan Sulfate from Monostroma nitidum
Source: Mar Drugs. 2021 Nov 30;19(12):685. doi: 10.3390/md19120685 (PMC8707894; doi:10.3390/md19120685)
Supplement: Supplementary file 1 [file marinedrugs-19-00685-s001.zip › marinedrugs-1477859-supplementary.pdf]

Anti-SARS-CoV-2 activity of rhamnan sulfate from *Monostroma nitidum*

Supplementary Data

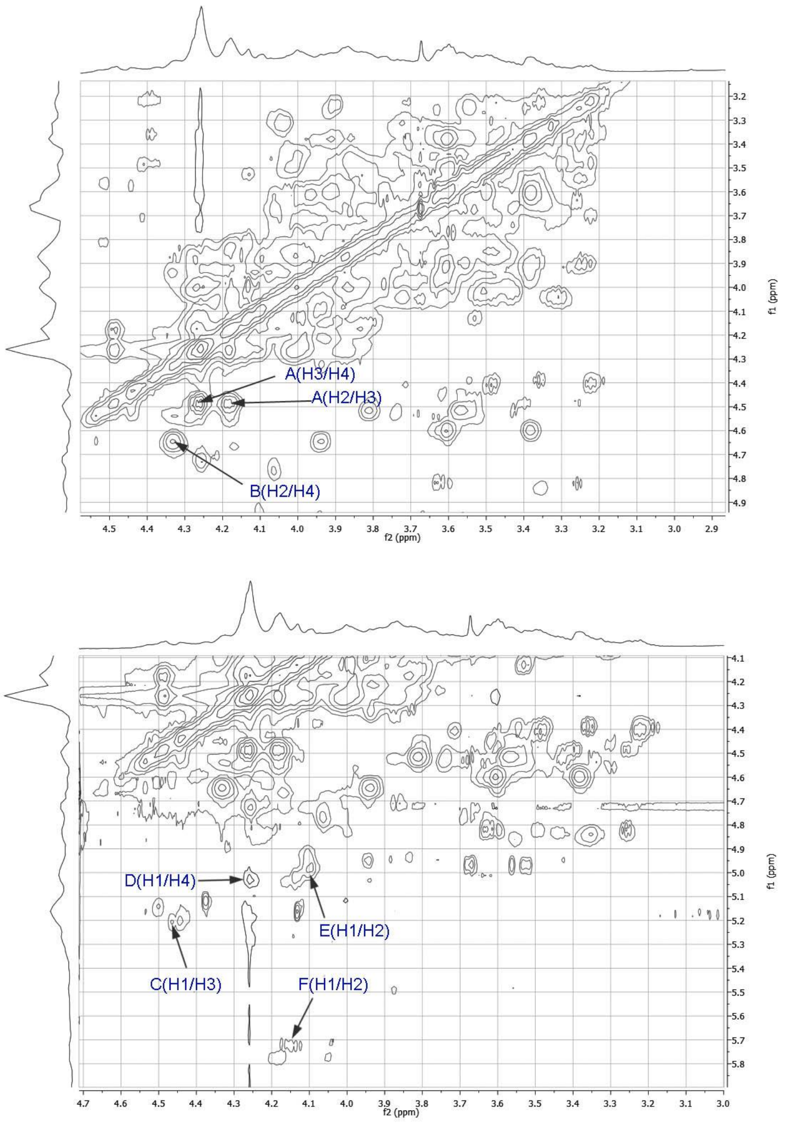

Figure S1 TOCSY spectrum of RS. A, α-L-Rha*p*3,4SO_3^-(1→; B, α-L-Rha*p*2,4SO_3^-(1→; C, →2)-α-L-Rha*p*3SO_3^-(1→; D, α-L-Rha*p*4SO_3^-(1→; E, →3)-α-L-Rha*p*4SO_3^-(1→; F: →3)-α-L-Rha*p*1SO_3^-
